# Eye vergence responses to novel and familiar stimuli in young children

**DOI:** 10.1016/j.actpsy.2019.01.007

**Published:** 2019-02

**Authors:** Flavia L. Esposito, Hans Supèr

**Affiliations:** a Department of Cognition, Development and Educational Psychology, Faculty of Psychology, University of Barcelona, Barcelona, Spain; b Braingaze SL, Mataró, Spain; c Neuroscience Institute, University of Barcelona, Barcelona, Spain; d Catalan Institution for Research and Advanced Studies (ICREA), Barcelona, Spain

**Keywords:** Short-term memory, Visual attention, Vergence, Eye movements, Childhood

## Abstract

Eye vergence is the slow movement of both eyes in opposite directions enabling binocular vision. Recently, it was suggested that vergence could be involved in orienting visual attention and memory having a role in cognitive processing of sensory information. In the present study, we assessed whether such vergence responses are observed in early childhood. We measured eye vergence responses in 43 children (12–37 months of age) while looking at novel and repeated object images. Based on previous research, we hypothesized that visual attention and Visual Short-Term Memory (VSMT) would be evidenced by differential vergence responses for both experimental conditions, i.e. repeated (familiar) vs. novel items. The results show that attention related vergence is present in early childhood and that responses to repeated images differ from the ones to novel items. Our current findings suggest that vergence mechanisms could be linking visual attention with short-term memory recognition.

## Highlights

• We measured eye vergence responses in children while looking at novel and familiar objects.
• Our results show that attention-related vergence is present in early childhood.
• The responses to repeated images are different than those to novel ones.
• Our observations suggest a role of eye vergence in attention and Visual short-term memory.

## 1 Introduction

The Visual Short-Term Memory (VSTM) system can create memory representations rapidly, based on object perception and mediated by visual attention (Hollingworth & Henderson, 2002; Johnson, Hollingworth, & Luck, 2008; Matsukura & Vecera, 2009). VSTM is needed to store perceptual information long enough so it can be integrated with new perceptual information (Hollingworth, Richard, & Luck, 2008), and it is used every time we blink, make a saccadic eye movement, compare objects, or when occlusion occurs (Hyun, Woodman, Vogel, Hollingworth, & Luck, 2009). Visual attention, which has a pivotal role in cognitive processing of sensory information, is a driver of VSTM (Astle & Scerif, 2011). It also strongly relates to recognition memory (Reynolds, 2015) where it can assist in encoding visual information into memory and influence already stored information (Griffin & Nobre, 2003).

Oculomotor structures form part of the attention circuits where saccadic eye movements reflect the outcomes of attention processing. Recently, it was suggested that besides conjugate eye movements, disconjugate or vergence movements play also a role in orienting visual attention (Solé Puig, Puigcerver, Aznar-Casanova, & Supèr, 2013; Solé Puig, Zapata, Aznar-Casanova, & Supèr, 2013). Vergence eye movements, where the eyes rotate in opposite directions, bring the two lines of sight to an intersection at a focus point in space. The inward rotation of the eyes is known as convergence, and the outward rotation of the eyes is known as divergence. During the process of orienting visual attention to a visual stimulus in the periphery, the eyes briefly converge. Such vergence responses were observed during top-down and bottom-up generated shifts of visual attention, where attentional load positively correlated with the strength of the vergence response (Solé Puig, Puigcerver, et al., 2013). A role of vergence in attention is supported by the observation of a correlation of eye vergence responses with the neural activity encoding shifts of visual attention (Solé Puig et al., 2016). Besides attention, success in memorizing objects was also associated with vergence responses (Solé Puig et al., 2015). Solé Puig and colleagues found that repeated stimuli elicited stronger vergence responses than novel ones. This was especially the case when the repeated stimulus was correctly identified. Based on these observations it was proposed that eye vergence could have a role in attention and memory processing of visual information.

The human binocular system, and thus the neural system for eye vergence, develop during the first few years of life (Jandó et al., 2012). Infants are typically born hyperopic (Mayer, Hansen, Moore, Kim, & Fulton, 2001), and with a narrow inter-pupillary distance (IPD; MacLachlan & Howland, 2002). A variety of behavioral and electrophysiological studies agrees that the onset of functional binocular interaction in human visual cortex normally occurs between 10 and 16 weeks of age in infants (Braddick, 1996). Visual sensitivity increases with cortical and foveal maturation in the first 6 months of life (Braddick & Atkinson, 2011). Visual maturation continues throughout early childhood, however binocular control may have a more extended developmental period and continues maturing during the following years of childhood (Jandó et al., 2012). If binocular control does not adequately develop, it may lead to a deficit in visual attention (Fawcett, Wang, & Birch, 2005). For example, children suffering from attention problems, like ADHD (Granet, Gomi, Ventura, & Miller-Scholte, 2005; Solé Puig et al., 2015; Varela Casal et al., 2018) and ASD (Milne, Scope, Pascalis, Buckley, & Makeig, 2009) have atypical or poor binocular control.

Visual attention and VSTM are already present right after birth and continue de develop during the early years of childhood (Courage & Howe, 2004; Rose, Feldman, Futterweit, & Jankowski, 1997; Rose, Feldman, & Jankowski, 2004). By 6.5 months, young children can form object representations, and they can use the features of those representations to individuate those objects—likely components of VSTM representations. Up to until 8 months, young children become able to store multiple items (Morgan & Hayne, 2006). Ross-Sheehy, Oakes, and Luck (2003), found preferences for changing displays at set sizes 2 and 3 in 10 and 13-month-old young children, indicating that they have a VSTM capacity that is sufficient to distinguish between changing and unchanging displays of arrays with up to 3 items. In adulthood, there may be a gender difference as men treat information differently (Bayliss, Pellegrino, & Tipper, 2005) than women, which have been found to perform better in episodic memory tasks involving face recognition (Yonker, Eriksson, Nilsson, & Herlitz, 2003) and object recognition (McGivern et al., 1998).

The ability to process and memorize visual information at early developmental stages implies that the underlying neural circuits are already formed by then and may even present sex variations. As eye vergence relates to and possibly has a role in attentional selection, we speculate that attention related eye vergence responses should be present at early stages, which may be different for girls and boys. To test this, we applied a recognition task that allowed discrimination between a familiar/repeated visual object and a novel object. This procedure has been shown its adequacy to test VSTM in children because it facilitates the comparison between concurrent displays, thereby increasing sensitivity to detect differences between the displays (Oakes & Ribar, 2005; Ross-Sheehy et al., 2003).

The results of our current study show that both repeated and novel images elicited vergence responses. Vergence responses to repeated images however are stronger than to novel images indicating a relation between vergence and visual memory. For both image types, pre-stimulus vergence responses were noticed where the strength was a function of presentation order. We suggest that the increased vergence responses reflect preparatory or attention processing during a VSTM task. Our current findings show attention related eye vergence already at early developmental stages and support a role of eye vergence in visual attention and memory.

## 2 Materials and methods

### 2.1 Participants

Young children were recruited from a regional public kindergarten. Forty-three young children, 12 to 37-month-old (26.75 ± 7.33); 14 girls (26.57 ± 6.56) and 29 boys (26.93 ± 8.11) composed the final sample. All subjects were born full-term, were in good health, and had neither visual nor neurological disorders. Participants with any accommodative problems, such as strabismus or nystagmus, were excluded from the sample. Written informed consent was obtained from their parents in accordance with the Helsinki Declaration. Participants were tested only after informed consent was given to and signed by their parents. The Ethics Committee of the University of Barcelona approved of the study.

### 2.2 Procedure

**Fig. 1:**
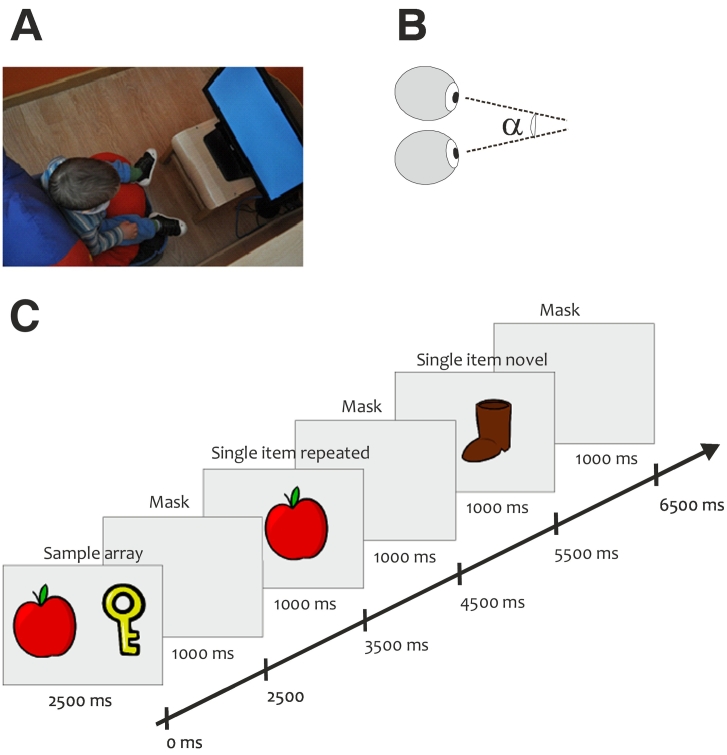
Experimental design A: Example of setup. B: Schematic explanation of the angle of eye vergence spanned by the visual angle formed by the right and left eye vectors. **C**: Task design scheme, adapted from a visual recognition memory paradigm. (For interpretation of the references to color in this figure, the reader is referred to the web version of this article.)

Young children were tested in a dimly lit room of the kindergarten. They sat comfortably in a car seat that was raised and lowered so as to standardize the position of each participant's eyes relative to the display monitor at approximately 60 cm distance of the eye tracker, surrounded by side panels to reduce visual distraction away from the monitor (see Fig. 1). Participants were not restrained in any way. The adult did not interact with the individual unless necessary to give reassurance. During testing, both experimenter and parent/caregiver were out of view from the child while able to monitor the child's gaze behaviour by means of a second screen.

The position of gaze was tracked using a binocular remote eye-tracking system at a sample rate of 30 Hz (X2-30 Tobii Technology AB, Sweden). The device was mounted beneath a 40 × 60 cm flat PC monitor with a display resolution of 1024 × 768 pixels, against a uniform grey background (50 cd/m^2^).

The equipment was calibrated at the beginning of each experiment. Calibration was done at 4 corner points plus a central point and was considered accurate with at least 1000 ms of gaze fixation to collect 30 gaze points at each calibration location within a radius of 50 pixels from the centre of the cartoon. After calibration was successfully completed, the task started. Each participant completed an average of 30 trials within approximately 3 min.

### 2.3 Visual presentation

Each trial started with the presentation of a sample array containing two-coloured cartoon images (approx. size 12° × 12°) of daily objects (see Fig. 1) presented for 2.5 s followed by a retention interval consisting of a grey mask for 1 s. After the mask ended, a sequence of two consecutive images (single-items) was presented. One of the objects belonged to the previous sample array and one was a completely novel object. Each object was presented for 1 s, followed by a grey mask of the same duration. The sequence of the single-items (repeated and novel images) was random. After every 6 trials the attention grabber, a looming cartoon with an accompanying sound to engage the participants' interest, was presented for 4 s. Each trial consisted of a new set of images. Therefore, none of the images were repeated across trials in order to target more specifically VSTM (Oakes, Baumgartner, Barrett, Messenger, & Luck, 2013; Ross-Sheehy et al., 2003).

### 2.4 Data preparation and statistical analysis

For gaze behaviour we calculated fixation duration and number. A fixation is considered when the change in eye position is less than 7 mm, lasting for at least 100 ms. We grouped participants in 4 age groups (12–18 months: 9 young children; 19–24 months: 9 young children; 25–30 months: 10 young children; 31–37: 15 young children) to have indications of possible developmental effects. The grouping criterion is based on previous research indicating that 1) the control of attention is not fully developed by 18 months (Rueda et al., 2004), 2) that the ability to overcome distracting information develops between 18 and 24 months (Clohessy, Posner, & Rothbart, 2001), and 3) that visual attention and VSTM become more flexible and more stable around 18–24 months old (Robinson & Pascalis, 2004).

Vergence responses were calculated by measuring the angle between the gaze vectors of both eyes using the dot product formula. For each eye, we used the 3D eye coordinate and 2D gaze coordinate provided by the Tobii X2-30 software to calculate the gaze vector. Vergence angle was then computed using the dot product formula of two gaze vectors by using the inverse cosine function. Due to poor quality, vergence data from 2 participants was excluded from the analysis. The raw signal was cleaned using the tracker's validity score (score of 1 to 4), which is a score that corresponds to every sample estimating the accuracy and quality of the recording. Low validity scores usually happen during saccades and blinks. Only maximum score (i.e. 4) lectures were left (approx. 80% of total per signal).

After computing the vergence responses, we took for each subject all vergence values of all trials per condition (repeated/novel), considering order of image presentation, within a time window of 2 s, from the onset of the mask that precedes the single-item probe. Finally, we calculated the mean vergence across subjects. Before averaging, the offset was removed, i.e. for each trial the average vergence responses during the first mask presentation were extracted from the time series to detect attentional related vergence response to the repeated and novel images. The obtained signal was then smoothed using a moving average with a 200 ms window. Vergence responses were averaged over a window of 400 ms prior to stimulus onset (pre-stimulus responses) and 400 ms after stimulus onset (post-stimulus responses). The same trials we used for vergence analysis were also used for pupil analysis. Before averaging, the offset was removed in the same way we did for the vergence responses and the signal was normalized by dividing it by its maximum.

For statistical analysis for vergence responses, we have applied a linear-mixed effect modelling approach. The primary analysis, to determine the effects of repetition, time window, order, age and gender on vergence responses, used a linear mixed model with repetition, time, order, actual age and gender as continuous or fixed factors and participant as a random factor. If memory is reflected in the vergence responses, we speculate that the type (repeated or novel) of the first image will affect the vergence responses to the second image and have an effect on vergence responses during the pre and post stimulus periods. We therefore assessed the interaction between image type and the order of presentation and time window. (Formula used: Vergence − 1 + Age + Gender + Order ∗ Type + Win ∗ Type; Model fit statistics: AIC: 10962, BIC: 11015, Log Likelihood: −5471.8, Deviance: 10944; 95% CIs). Statistics with a value of p < .05 were considered significant. Vergence responses of all trials were also analysed per time sample using a *t*-test with a significance level of p = .05 (e.g. Solé Puig, Puigcerver, et al., 2013). These results are presented within the figures. Eye fixation behaviour and behavioral responses were analysed using a *t*-test. Software written in Python and Matlab (R2013b & statistical toolbox) was used for eye data and statistical analyses.

## 3 Results

### 3.1 Gaze fixations

**Fig. 2:**
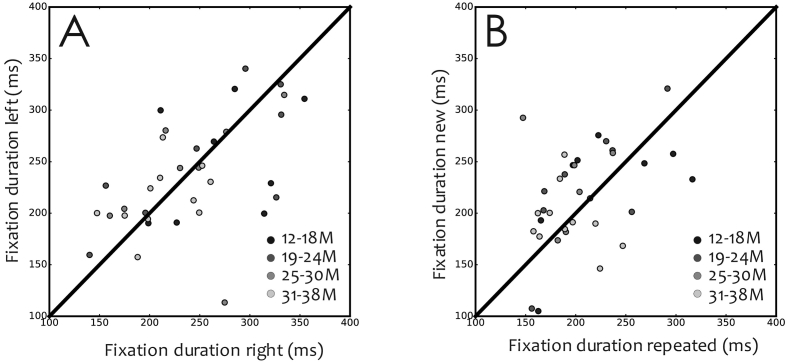
Mean fixation duration per subject. Different grey-scale (from light grey to black) colours were used to group each subject according to age group (12–18 months old, 19–24 months old, 25–30 months old, 31–38 months old). A: Average fixation duration when looking at the right image of the stimulus pair versus the fixation duration when looking at the left image. B: Average fixation duration for repeated versus new images. Diagonal lines represent unity.

To assess whether the duration or number of fixations impacted performance for repeated or novel presentations of single items, we analysed fixation behaviour during the sample array (i.e. the simultaneous two-coloured cartoon images) separately for the to-be-repeated image and the not-to be repeated image depending on display side (left/right from midline). The results show (Fig. 2) that average number of fixations (mean ± std.: 1.12 ± 0.80) on the side corresponding to the to-be-repeated image was similar to the number of fixations on the side of the not-to-be-repeated image (mean ± std.: 1.16 ± 0.94). The average fixation duration on the side corresponding to the to-be-repeated image (mean ± std.: 237.08 ± 51.67 ms) was neither statistically different from the fixation duration on the side of the not-to-be-repeated image (240.88 ± 57.97 ms). Overall, when pairs of images were presented simultaneously in the sample array, children showed similar duration and number of fixations per corresponding display side of the screen.

The average duration and number of fixations were also calculated for the novel and repeated images separately (single-item probe). The average fixation duration was 224.61 ± 115.52 ms (mean ± std) for novel images and 250.78 ± 122.39 ms (mean ± std) for repeated images (Fig. 2). The difference was not statistically significant (p = .37). Neither the number of fixations turned out significantly different (mean ± std; repeated images, 1.39 ± 0.32; novel images, 1.40 ± 0.32; p = .25).

We were also interested in knowing whether there is an effect due to the order of image presentation. We found that the number of fixations to the repeated item depended on whether it appeared on the first or second order of image presentation. When the repeated image was presented first (repeated 1st) the average fixation number (mean ± std: 1.36 ± 0.35) was reduced (p < .05) compared to the fixation number to the repeated image (mean ± std: 1.42 ± 0.33) when presented second (repeated 2nd). The average fixation number did not turn out to be significantly (p = .45) different in the novel condition between order of presentation (novel 1st; mean ± std: 1.38 ± 0.36; novel 2nd; mean ± std: 1.42 ± 0.33). The average fixation duration to the repeated item was not significantly (p = .45) different when the repeated item was presented first (repeated 1st; mean ± std: 231.73 ± 125.07 ms) than when presented second (repeated 2nd; mean ± std: 264.43 ± 127.07 ms). This was also true for the novel items (novel 1st; mean ± std: 214.55 ± 100.37 ms; novel 2nd; mean ± std: 236.72 ± 130.81 ms; p = .20).

### 3.2 Vergence responses to novel and repeated stimuli

**Fig. 3:**
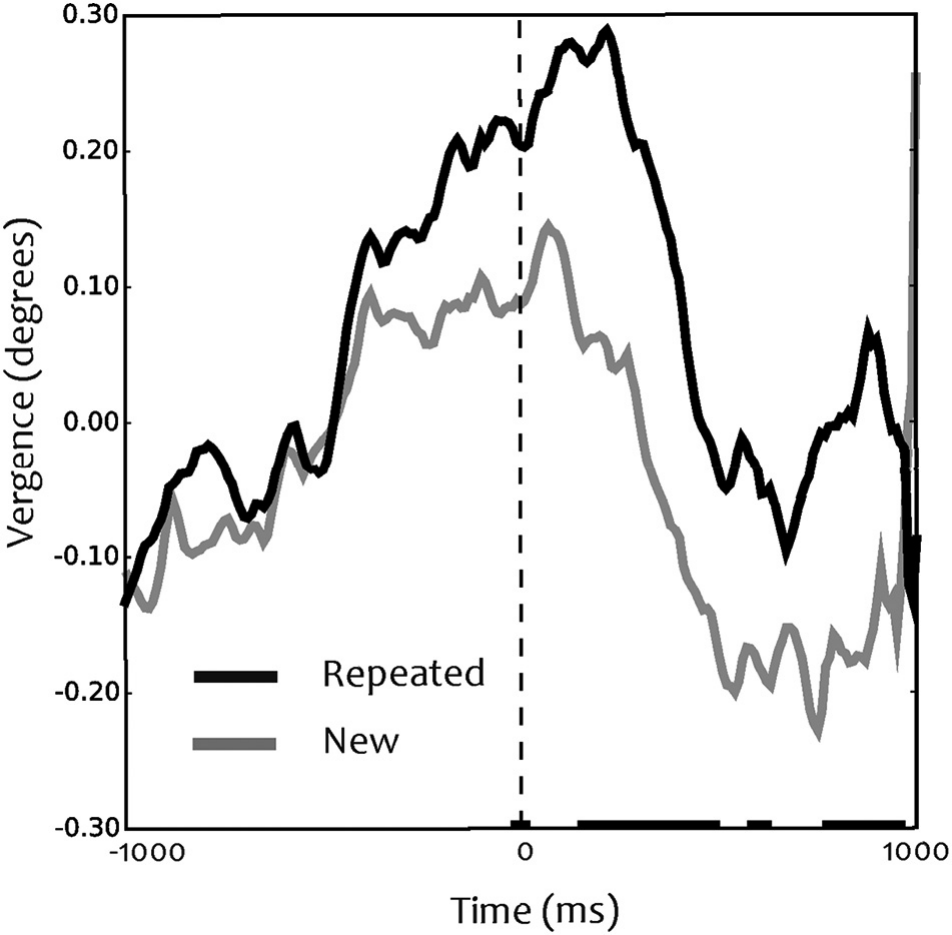
Vergence eye movements. Mean vergence responses to repeated (familiar) and novel items. Vertical dotted line depicts the onset of the image. Time is from stimulus onset. The lines at the bottom depict the time samples when the vergence angle significantly (p < .05) differs between repeated and novel conditions.

Next, we analysed the angle of eye vergence while children looked at the novel and repeated images (single-item probe). A convergence response was observed, which started around 500 ms prior to the onset of the single item and reached a maximum of 0.1–0.3° around stimulus onset. (Fig. 3). Around 500 ms after stimulus presentation, the eyes started to diverge towards baseline level. The increase in the angle of eye vergence occurred for repeated and novel images.

During the image presentation (after image onset), the average vergence response was stronger to repeated images that the vergence response to novel ones (Fig. 3; see black horizontal lines at the x-axis). The model revealed a significant effect of image type on vergence responses (*t* = −2.38, df = 2797, p = .017, CI = −1.22, −0.12). In addition, the factor ‘age’ as rendered by the model calculations was significant on vergence responses (*t* = 5.00, df = 2797, p = 5.9e-07, CI = 0.01, 0.03), although the effect of gender type on vergence responses was not significant (*t* = 1.4078, df = 2797, p = .16, CI = −0.03, 0.21).

**Fig. 4:**
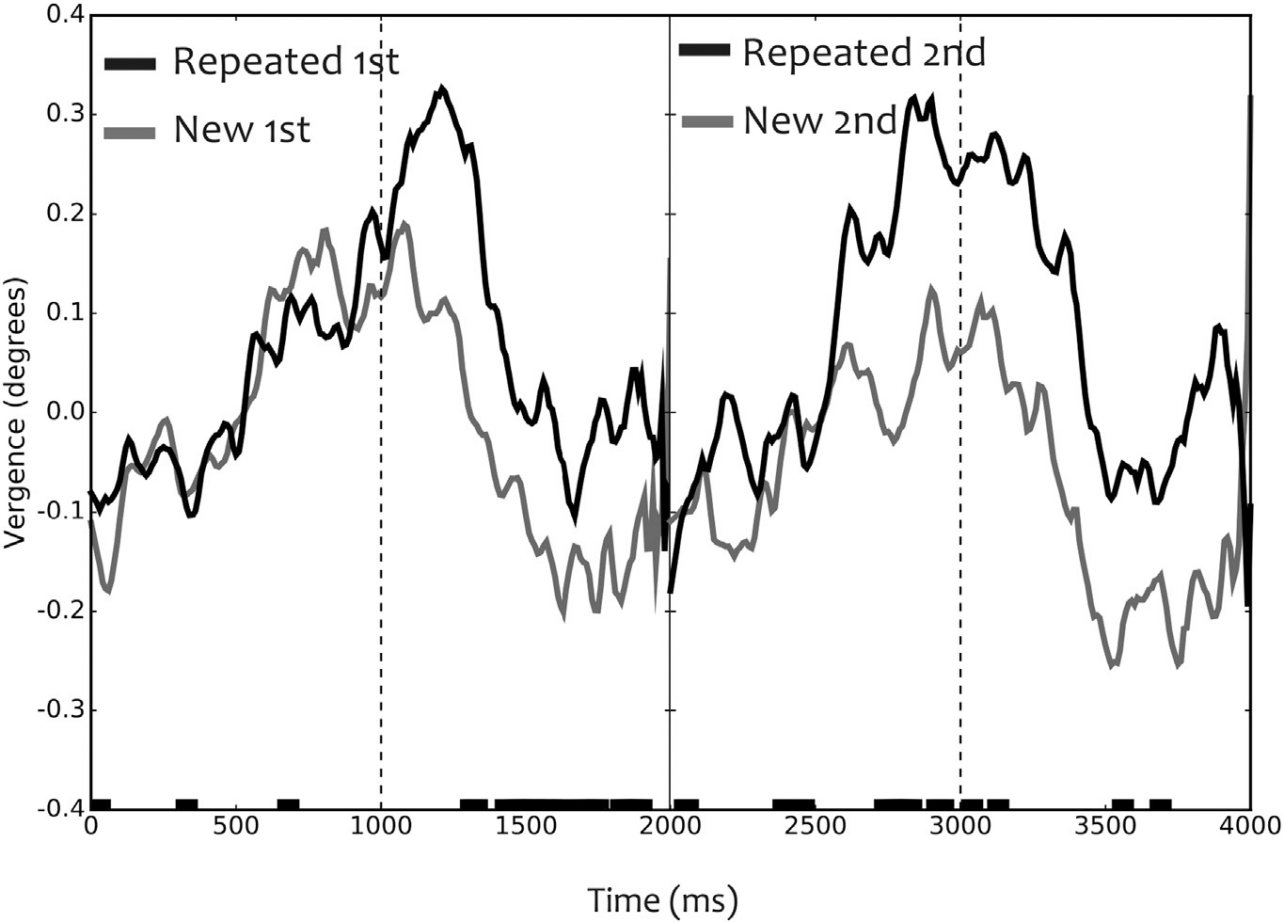
Vergence responses during the trial. Vergence responses to first and second image presentation. Vertical dotted lines depict the onsets of the images. Time is from start of the trial (from mask onset 0–1000 ms; from stimulus onset (random single item probe) at 1000–2000 ms; mask from 2000 ms–3000 ms and stimulus onset from 3000 to 4000 ms). The lines at the bottom depict the time samples when the vergence angle significantly (p < .05) differs between repeated and novel conditions.

We then analysed the vergence responses to repeated and novel images as a function of the order of stimulus presentation. We therefore separately calculated the vergence responses to the first and second image. For both first and second images, a pre-stimulus vergence response was observed. The strength of the pre-stimulus responses to novel as well as to repeated images was similar when they were presented as the first image (Fig. 4). However, the strength of the pre-stimulus vergence response to the second stimulus was stronger if the image was a repeated one (Fig. 4; see black horizontal lines at the x-axis). The post stimulus responses were stronger for repeated stimuli (Fig. 4). The model revealed no significant interaction between vergence responses with presentation order (*t* = 0,20, d = 2797, p = .84, CI = −0.22, 0.27). In spite of this, the model did show a significant effect of image type on time window (pre/post stimulus) condition (*t* = 2.52, d = 2797, p = .01, CI = 0.07, 0.57).

### 3.3 Pupil size

**Fig. 5:**
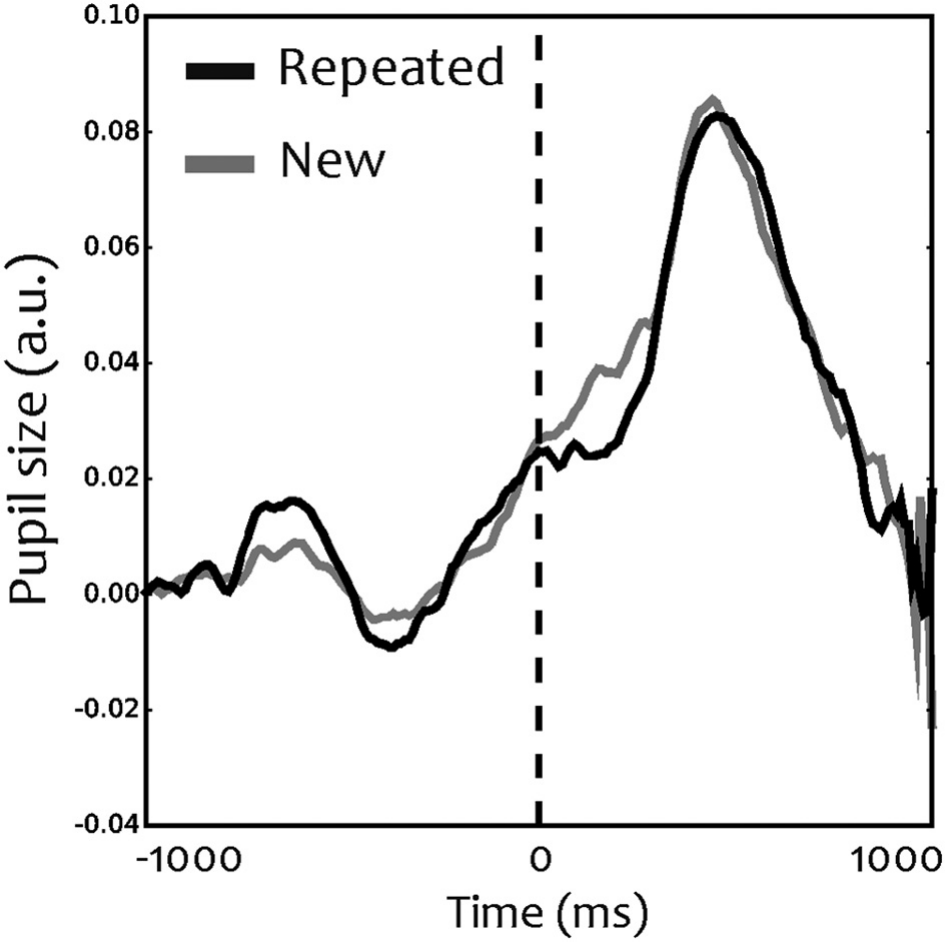
Pupil size. Pupil responses to repeated and novel items. Vertical dotted line depicts the onset of the image. Time is represented previous to and from stimulus onset No samples where pupil responses differed significantly between conditions were detected.

Changes in pupil size are associated with attentive processing (Porter, Troscianko, & Gilchrist, 2007). We therefore analysed the changes in pupil diameter. For both repeated and novel items pupil began dilating around 300 ms before stimulus onset, reaching peak values at approximately 400 ms after presentation of the single item. The results on the modulation in pupil size show that pupil size is similar for repeated images and novel items (Fig. 5).

## 4 Discussion

In this study, we assessed eye vergence in children while looking at novel and repeated object images. We show that the presentation of an image elicits a vergence response, which is stronger when the image is a repeated one. VSTM is present right after birth and from early childhood, as children can distinguish between changing and unchanging displays (Courage & Howe, 2004; Rose et al., 1997; Rose et al., 2004). We therefore argue that the observed differential vergence responses between repeated and novel stimuli reflect VSTM. This idea agrees with the reported observation of vergence responses to face stimuli in young children (Esposito & Supèr, 2018), and with the finding of vergence responses to correctly remembered images in adults (Solé Puig et al., 2015).

We found no clear differences in duration nor frequency of fixations, for neither repeated nor novel items. We only observed a reduced number of fixations to the repeated objects when presented first. This may suggest that children did not display any initial preference for location (left or right side of the screen), or object type (repeated or novel objects). However, looking preferences at population level may appear to be random despite individual infants showing clear familiarity or novelty preferences (Bogartz & Shinskey, 1998). In addition, models of infants' attentional preferences such as Hunter and Ames' (1988) show that random looking behaviour should not be equated with a failure to discriminate. Moreover, individual infants pass through a period between preferring familiarity and preferring novelty when both attract their attention equally, which will appear as random looking (see also Roder, Bushnell, & Sasseville, 2000).

Changes in pupil diameter provide a momentary, involuntary and unbiased measure of arousal and cognitive load (Karatekin, 2007; Porter et al., 2007; Rosa, Oliveira, Alghazzawi, Fardoun, & Gamito, 2017). We observed that stimuli evoked modulation in pupil size for repeated and novel images. In contrast to vergence responses, pupil responses to repeated and novel stimuli did not differ. Moreover, they showed a different temporal pattern than the one present in vergence responses. A difference in response patterns between pupil and vergence has been previously reported (Solé Puig et al., 2015; Solé Puig, Zapata, et al., 2013). Thus, even though the neural mechanisms that control vergence and pupil size are linked, the attention related vergence responses cannot solely be explained by changes in pupil size.

Our finding of vergence responses during the mask period, prior to the presentation of the repeated or novel image could represent an element of anticipation or expectancy once children infer the sequence due to the format of serial repetitions (Fiser & Aslin, 2002). Anticipatory vergence responses also occur prior to behavioral responses in a memory task, which are stronger when the responses are correct (Solé Puig et al., 2015). Moreover, the induced vergence responses when orienting visuospatial attention after cueing (Solé Puig, Zapata, et al., 2013) can be considered as some kind of a preparatory phase for subsequent processing the target stimulus.

A role of attention in VSTM tasks has been demonstrated in adults (e.g., Rudkin, Pearson, & Logie, 2007; Salway & Logie, 1995) and in infants (e.g., Ross-Sheehy, Oakes, & Luck, 2011). Attention may be a vehicle by which information is stored in memory (Schmidt, Vogel, Woodman, & Luck, 2002). Yet, little is known about the principles that govern the attentional process required for perception and memory. Reynolds, Courage, and Richards (2010) showed that the cortical areas controlling attention to stimuli may be similar to cortical areas controlling recognition memory. The conceptualization of VSTM control as equivalent with visual attention forms part of a larger claim that VSTM and attention are simply two terms to describe the same selective mechanism (Chun, 2011; Cowan, 2001; Gazzaley & Nobre, 2012; Kiyonaga & Egner, 2013; Theeuwes, Belopolsky, & Olivers, 2009; Wheeler & Treisman, 2002). The nuclei that are responsible for vergence eye movements receive direct input from cortical areas involved in attention control. Therefore, attention, vergence and VSTM circuits appear to be coupled, indicating shared neural control mechanisms.

The function of vergence in attention processing remains unclear. However, it could be hypothesized that eye vergence may have a role in cortical synchrony (Super, van der Togt, Spekreijse, & Lamme, 2003; Van Der Togt, Kalitzin, Spekreijse, Lamme, & Supèr, 2005). At a neural level, attention is characterized by a change in correlated activity which starts just before stimulus onset and lasts for a few hundred ms after stimulus onset (Super et al., 2003; Van Der Togt et al., 2005). Usually, both eyes move in similar directions and thus operate in a coupled manner. Vergence is an eye movement where eyes move in opposite directions and thus represents an uncoupling. In that sense, the change in eye motion mimics the change in neural correlated activity patterns promoting cognitive processing of sensory information.

To our knowledge, this is the first study assessing attention related eye vergence responses in a VSTM task in young children. Children showed differential vergence responses while looking at a familiar vs. novel visual stimulus. The current findings therefore suggest eye vergence responses related to attention and memory are present at early developmental stages. This finding may be important for understanding certain neurodevelopmental disorders.
